# A Unique Case of Sporadic Optic Pathway Glioma in an Infant With Acute Nystagmus

**DOI:** 10.7759/cureus.17568

**Published:** 2021-08-30

**Authors:** Colton T Knight, Hunaid N Rana, Todd Standley

**Affiliations:** 1 Radiology, University of South Alabama, Mobile, USA

**Keywords:** optic pathway glioma, optic nerve infiltration, neurofibromatosis 1, nf-1, nystagmus, sporadic opg

## Abstract

Optic pathway gliomas (OPGs) are a classic pathology seen in patients with neurofibromatosis I (NF-1); however, they are frequently seen as sporadic masses in patients with mutations activating the mitogen-activated protein kinase (MAPK) pathway. These sporadic tumors present rapidly with vision deficits, compared to those in neurofibromatosis I, which may be found incidentally. They can involve multiple aspects of the optic pathway and have classic imaging findings that make definitive diagnosis possible with magnetic resonance imaging. This case highlights a six-month-old boy who had an acute history of nystagmus and severe milestone regression, who was diagnosed with bilateral optic pathway gliomas. This case describes the associated imaging findings in addition to a discussion of management and overall prognosis of sporadic compared to NF-1-associated optic pathway gliomas.

## Introduction

Optic pathway gliomas (OPGs) are a low-grade malignancy of the optic nerve and associated tracts. These tumors compose 3-5% of all pediatric CNS tumors with 71% identified in the first decade of life [1].^ ^The presentation of symptoms is classically vision loss, with field defects dependent on the location and size of the tumor. These tumors usually involve the optic chiasm with further extension to the hypothalamus, thalamus, or third ventricle, with only 25% involving the optic nerve alone [2].

Classically, optic pathway gliomas are associated with neurofibromatosis I (NF-1). The incidence of NF-1 in patients with OPGs ranges widely from 10% to 70% across cross-sectional studies, with a mean incidence of 29% [3]. When NF-1 is involved, there is a signal activation of the RAS pathway through inactivation of neurofibromin 1, leading to tumor cell proliferation. On the other hand, in sporadic OPGs, the most common gene mutation is a duplication of the BRAF kinase domain, leading to a BRAF-KIAA1549 fusion gene and subsequent mitogen-activated protein kinase (MAPK) pathway activation [4]. Though these tumors have low-grade histology, their management can be challenging due to limited surgical intervention, and their spread can be aggressive, making early diagnosis through imaging vitally important.

## Case presentation

A six-month-old Caucasian male with no significant past medical history presented to the pediatric emergency department for a chief complaint of frequent unusual eye movements as reported by the parents. The patient began having intermittent rotary nystagmus approximately three weeks ago, and since then it progressed and became more continuous, worsened by changes in position or flashing lights from toys brought in his visual field. The parents also noticed the patient sleeping more than usual, only staying awake for an hour at a time, and had a subsequent severe regression in milestones, with an inability to roll over or sit unsupported.

**Figure 1:**
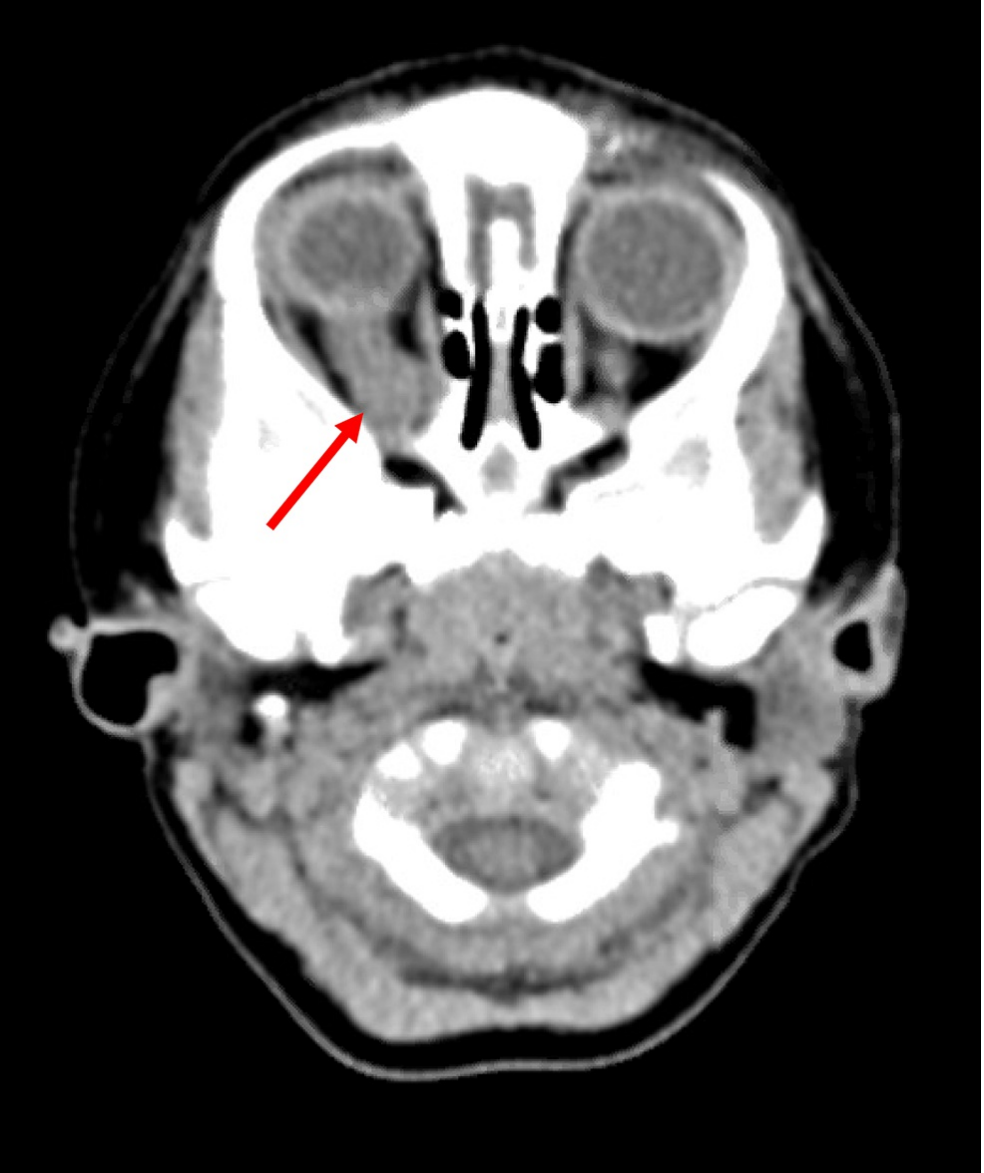
Noncontrasted axial CT of the brain with images centered through the optic nerve demonstrating diffuse thickening with mass-like appearance of the right optic nerve. There is also a thickening and mass-like appearance of the left optic nerve.

Initial workup in the emergency department included a CT brain without contrast, which showed evidence of thickening of the bilateral optic nerves with associated masses and diffuse bilateral ventriculomegaly (Figure 1). This finding was concerning for possible bilateral optic pathway gliomas, at which point a dedicated MRI of the brain and orbits was recommended, along with further genetic workup for possible neurofibromatosis I.

**Figure 2:**
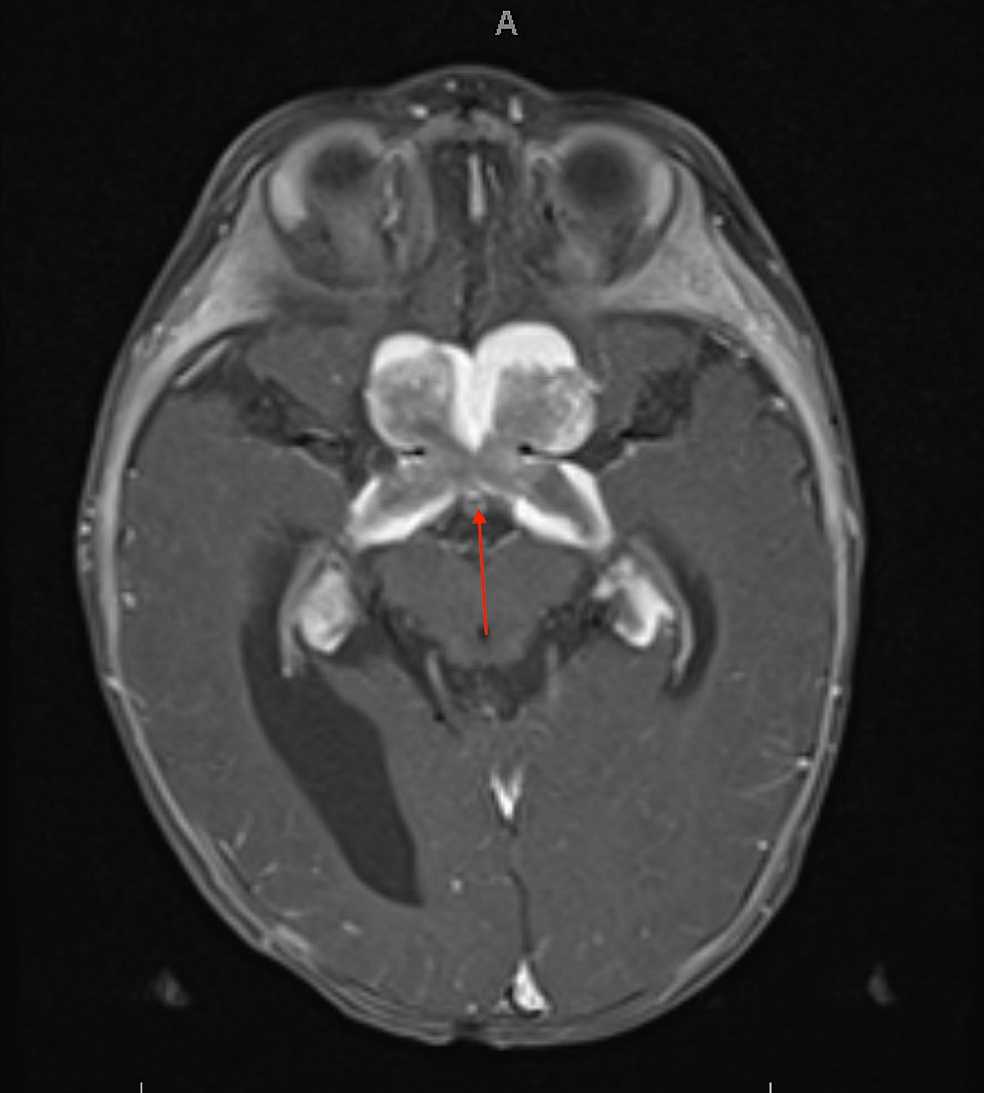
T1 postcontrast axial image centered through the optic chiasm demonstrates large enhancing mass in the optic chiasm extending into the bilateral lateral geniculate nuclei within the thalamus.

**Figure 3:**
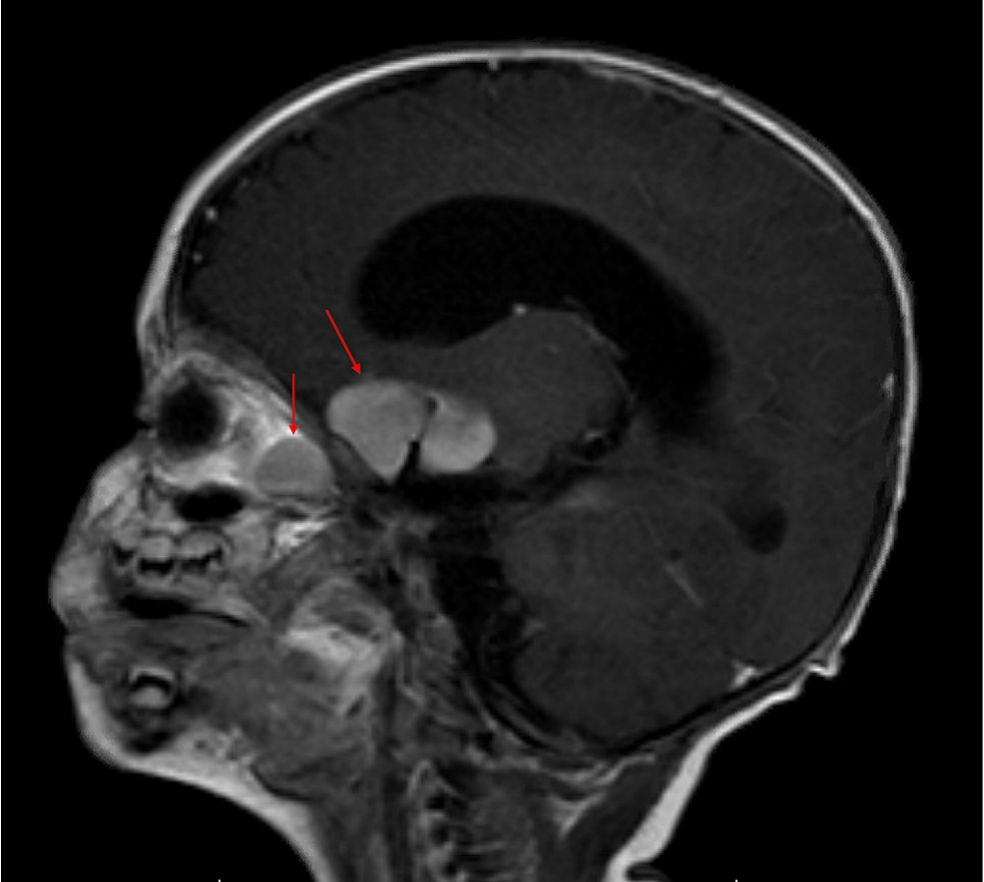
T1 postcontrast sagittal image centered to the right optic nerve demonstrates a diffuse enlargement of the optic nerve with extension along the optic tract and optic chiasm. Flow void in the center of the mass is consistent with encasement of the supraclinoid internal carotid artery on the right.

MRI of the brain and orbits confirmed these findings, showing diffuse enlargement of the optic tracts extending from the lateral geniculate nuclei to the optic chiasm and into the bilateral optic nerves, with a mass demonstrating 270º encasement of the bilateral supraclinoid internal carotid arteries and near circumferential encasement of the bilateral A1 segments of the anterior cerebral arteries (Figures 2, 3).

These imaging findings confirmed the suspicion of bilateral optic pathway glioma, and as a result, the patient was admitted to the hospital and evaluated by both neurosurgery and pediatric hematology-oncology. Neurosurgery did not recommend any surgical intervention at the time, and the hematology-oncology team recommended proceeding with chemotherapy. His genetic testing results showed no evidence of clinically significant variants of neurofibromatosis I or II, suggesting a sporadic bilateral optic pathway glioma. The patient was then discharged and was followed up on an outpatient basis by both ophthalmology and pediatric hematology-oncology for maintenance chemotherapy with carboplatin and vincristine.

## Discussion

Sporadic optic pathway gliomas arise from astrocyte proliferation in the optic pathway, oftentimes from a mutation in the BRAF-kinase pathway. As a result of this proliferation, a phenomenon known as arachnoidal hyperplasia occurs, with astrocytes extending through the layers of the optic tract into the subarachnoid space leading to meningeal cell and ultimately tumor cell proliferation [5]. These tumors can grow significantly through this mechanism, leading to an early mass effect and subsequent vision changes.

The typical constellation of symptoms seen in children with sporadic optic pathway gliomas usually includes loss of vision, proptosis, nystagmus, hormonal deficits from hypothalamic involvement, and hydrocephalus. These sporadic OPGs display vision loss in up to 75% of cases [6]. They inherently carry a more complex management strategy as the growth is more invasive when compared to NF-1 associated OPGs.

Imaging with MRI and CT provide the best non-invasive method for definitive diagnosis of OPGs. Prior to the advances in imaging, biopsies were routinely performed on these tumors, but this procedure was invasive, and the histopathologic samples rarely informed specific treatment for these tumors. On CT, there is a typical finding of diffuse enlargement of the optic nerves, appearing isodense, and rarely demonstrating calcification within the nerve. Fine details of the spread of the glioma are hard to appreciate on CT though, so it is recommended to perform an MRI with and without contrast of the brain and orbits. In T1-imaging, the glioma appears isointense relative to the optic nerve, and there is a clear appearance of the outline and size of the mass. In T2-imaging, the mass appears hyperintense giving a better indication of surrounding invasion [6].

OPGs can be found at different anatomic locations along the optic pathway, and these have varying appearances on imaging. NF-1 tumors frequently involve the optic nerve, with a circumferential enlargement of the nerve with elongation and kinking. Alternatively, in sporadic tumors, there may be a mass in the optic chiasm. Some are large enhancing masses with exophytic components, while others will appear as a non-enhancing chiasmatic enlargement. Cystic areas are seen frequently in sporadic tumors, while this is a very uncommon finding in NF-1. They may also extend beyond the regions of the optic pathway, most frequently to the hypothalamus. These tumors are commonly classified using the Dodge Classification System first proposed by Dodge et al. in 1958, dividing the tumors by anatomical location [7]:

Stage 1: Optic nerves only.

Stage 2: Chiasm involved.

Stage 3: Hypothalamic involvement and/or adjacent structures.

Management of patients diagnosed with OPGs initially involves deciding between observation compared to intervention with surgery, radiation, or chemotherapy. Observation is only considered due to the recognized phenomena of spontaneous regression of OPGs in NF-1-associated tumors. Generally, intervention is recommended if there are any visual or neurological symptoms. Prior to the advent of modern imaging, debulking surgery was frequently performed, but these aggressive interventions have since been reconsidered due to the risk of damage to parenchymal and vascular structures near the optic tracts. Radiotherapy has also been used for the management of these tumors, but despite advances in three-dimensional (3D) mapping to avoid extensive irradiation of normal tissue, due to the nearby vascular structures of the circle of Willis, there is a risk of associated vasculopathy and late neurological deficits. Given these long-term effects, chemotherapy has been established as the mainstay of treatment, using combination therapy with carboplatin and vincristine or the thioguanine, procarbazine, lomustine, and vincristine regimen [1]. Overall, the long-term outcomes for patients with sporadic OPGs are variable, but studies published by Wan et al. suggest as high as 75% of patients have persistent long-term visual loss despite treatment [8]. Overall survival is generally high though, with studies ranging between 92% to 98% [9].^ ^Thus continuous ophthalmological follow-up and MRI surveillance remain important in these patients to monitor for progression.

## Conclusions

We highlight a unique case of a young patient who presented with nystagmus and regression of milestones, who was found to have a stage 3 optic pathway glioma as confirmed by MRI of the brain. This case emphasizes the differences seen between sporadic optic pathway gliomas compared to those associated with neurofibromatosis I. The diagnosis can be made definitively via MRI, avoiding an invasive and potentially dangerous surgical biopsy. These patients can be further managed with chemotherapy and consistent evaluation with a multidisciplinary team of ophthalmologists, neurologists, and pediatric hematologist-oncologists, in addition to consistent MRI surveillance if there is evidence of neurological or vision change.
